# Demographic dividend-favorable policy environment in two pre-dividend African nations: review of national policies and prospects for policy amendments in Nigeria and Tanzania

**DOI:** 10.1186/s12889-023-15690-z

**Published:** 2023-06-05

**Authors:** Xiaomeng Chen, Neia Prata Menezes, Jean Christophe Rusatira, Carolina Cardona, Mojisola Odeku, Deanna Kioko, Jessica Castro, Charity Ibeawuchi, Joel Silas Lincoln, Deo Ng’wanansabi, Jacob Macha, Abubakar Msemo, Nazir Yusuph, Jose G. Rimon

**Affiliations:** 1 Johns Hopkins Bloomberg School of Public Health, Baltimore, MD USA; 2 Center for AIDS Prevention Studies, University of California, San Francisco, San Francisco, CA USA; 3 Centre for Communication Programs Nigeria, Lagos, Nigeria; 4 Tanzania Communication and Development Center, Dar Es-Salaam, Tanzania

**Keywords:** Demographic dividend, Development policy, Demographic transition, Economic development, Sub-Saharan Africa

## Abstract

**Background:**

In collaboration with local partners, we reviewed 18 national policy documents across two sub-Saharan African countries identified as pre-dividend nations by the World Bank in 2017: Nigeria and Tanzania. Our aim was to assess national policies in pre-dividend countries and to determine whether national strategies were primed to capitalize on changing demographic structures, maximally attain the demographic dividend, and augment socio-economic growth.

**Methods:**

We conducted policy reviews by focusing on five key sectors of the Gates Institute Demographic Dividend Framework: Family Planning, Maternal and Child Health, Education, Women’s Empowerment, and Labor Market. This framework was developed as a tool for countries to apply targeted policies for accelerating the demographic dividend based on their demographic structure. For each component we used a comprehensive list of indicators, defined via a systematic literature review, through which we assessed national policies aimed at maximizing the demographic dividend.

**Results:**

Between the two countries, we observed persistent gaps in policies targeting family planning. Although more comprehensive, policies addressing maternal and child health, education, women’s empowerment, and labor market still lagged in their specificity and measurability. We identified specific policy amendments and alternatives that Nigeria and Tanzania could consider to mitigate these gaps. We also stress the importance of designing measurable policy initiatives across sectors.

**Conclusions:**

Based on these recommendations, as Nigeria, Tanzania, and other pre-dividend nations start experiencing rapid demographic changes, they may consider implementing routine policy reviews to strengthen policies across the five key sectors and harness the benefits of a demographic dividend.

**Supplementary Information:**

The online version contains supplementary material available at 10.1186/s12889-023-15690-z.

## Introduction

In 2017, the African Union committed to “Harnessing the Demographic Dividend Through Investments in Youth”, prompting leaders across the continent to develop multisectoral policy roadmaps and action plans for attaining the demographic dividend [1]. The demographic dividend presents countries with the opportunity to accelerate economic growth and maximize per capita gains [2–4]. These benefits come to fruition because of a demographic transition initiated by declines in national fertility and infant and child mortality rates. These changes work in tandem to modify the population age structure, limiting the number of child dependents and increasing the number of working age adults, resulting in a lowered dependency ratio and a larger labor force [5]. With a larger workforce, countries have the potential to boost their economic output and work to impart social and economic change.

However, the demographic transition and subsequent dividends in economic growth are not automatic. Indeed the extent to which countries benefit from this demographic transition is highly dependent on the existence of policies that establish optimal conditions for capturing the full returns of the demographic dividend [2, 3, 6]. Moreover, countries have a limited window of opportunity to act on maximizing the potential of the demographic dividend. Reher [7] estimated the window of opportunity was open for 100 years for Spain and Sweden and only 10 to 30 years for a subset of developing countries. Eventually, the demographic age structure will shift again when the increased adult population ages and enters an older, less-productive cohort that relies on their savings and retirement funds [8, 9]. In conjunction with smaller birth cohorts due to fertility declines, the dependency ratio will subsequently rise again, but will be characterized by the need to care for elderly populations. Evidence suggests that ensuring demographic dividend-favorable policies are in place, preferably before entering the demographic transition, can assist countries to fully harness the benefits of the demographic transition. However, most of this evidence comes from Southeast Asian and European countries with limited knowledge from African countries [2, 3, 10].

Bloom and colleagues have previously described that investments in health, education, and the labor-market are key to capitalizing on the demographic transition [4]. More specifically, key policy areas should: expand family planning and reproductive health to facilitate rapid fertility declines; improve maternal and child health and nutrition to reduce child mortality; increase educational attainment to facilitate a strong labor force and productivity; and invest in the labor and economic sectors to promote job creation and economic growth [2, 3, 11, 12]. Less well-documented are the specific policy investments and strategies that countries should enact to create a demographic dividend-favorable environment.

The Population Reference Bureau (PRB) recently conducted a literature review to identify specific policy inputs conducive to achieving the demographic dividend [13]. This review provided evidence that policy interventions like legalizing contraception, establishing family planning service delivery and outreach, investing in disease prevention and public health, offering free public education, reinvesting gross domestic product (GDP) growth in working-age populations, developing open trade policies, and encouraging foreign direct investment, among others, can facilitate attainment of the demographic dividend. Similarly, the World Bank reviewed policy documents for policies that would catalyze and accelerate a demographic transition among eight West African countries [14]. Their review documented achievements and opportunities for improvement in allocating financial resources, stakeholder support, institutional building and strengthening, and results monitoring to fast-track the demographic dividend.

Although knowledge of evidence-based policies to realize the demographic dividend exists, the extent to which they are incorporated into national planning is not well understood. Discerning the degree to which these policies are embedded within policy frameworks requires a comprehensive review of the national policy landscape. This intensive process can be accomplished through an exhaustive review of national strategic frameworks and sector-specific policy documents. However, the scope and relevance of these policies may vary depending on which phase of the demographic transition a country is experiencing. For instance, most countries in sub-Saharan Africa are in the pre-demographic dividend phase, with total fertility rate (TFR) greater than four children per woman, or the early-demographic dividend phase, whereby TFR is below four children [15].

Evidence suggests that pre-dividend countries should prioritize human development outcomes, with a goal towards accelerating the fertility decline, creating a population age structure with fewer dependents, and assisting a growing proportion of the population that is working age to develop the necessary skills to enter the labor market in the future. Conversely, early-dividend countries should prioritize accelerating job creation to ensure that the growing share of the working-age population remains employed to promote economic growth. The labor market must develop productive jobs to appropriately match labor supply with a growing labor demand. Regardless of demographic transition stage, countries adopt a multisectoral and integrated approach to ensure that the right supportive policies are in place to catalyze the demographic transition and translate it into concrete economic growth.

While studies have suggested specific policy enactments for countries to capitalize on changing demographic structures, these suggestions were mostly generated from the experiences in Southeast Asian and European countries. Furthermore, there are no demographic dividend-favorable policies specific to stages of the demographic transition in African countries in the existing literature. Using the Demographic Dividend Framework, developed by the Gates Institute, we aimed to review the policy landscape of two pre-dividend African nations as case studies, and to identify existing evidence-based policies conducive to fostering a demographic dividend-favorable environment in the pre-dividend context of each country. We also assess existing national policies based on their degree of comprehensiveness and identify gaps. We subsequently make recommendations regarding policy amendments that both African nations could undertake to capitalize on their changing demographic structures to attain a demographic dividend, and boost their economies.

## Methodological approach

### Conceptual framework

**Fig. 1:**
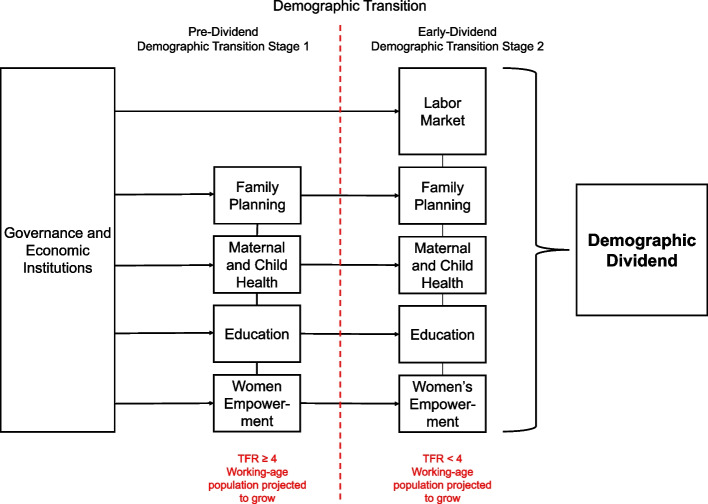
The Gates Institute Demographic Dividend Framework

The current stage of a country’s demographic transition largely influences how and why decision-makers prioritize the needs of the populations they serve. The Gates Institute developed the Demographic Dividend Framework (Fig. 1) to highlight essential country-wide sectors to focus policies for attaining a demographic dividend [16]. These sectors are divided into six key factors: i) Governance and Economic Institutions, ii) Family Planning, iii) Maternal and Child Health, iv) Education, v) Women’s Empowerment and vi) Labor Market. These sectors are stratified by demographic typology as defined by the World Bank in 2017: pre-dividend and early dividend [17–19]. Early-dividend countries have a TFR below four children per woman; while pre-dividend countries have a TFR greater or equal to four. Both pre-dividend and early-dividend countries are expected to increase the working age population share until 2030. Labor Market is added as a sixth sector for early dividend countries but not for pre-dividend. Since early dividend countries have already embarked on a fertility decline, in this stage they need to create productive jobs to absorb the large youth cohort that is entering the labor market. This policy review focuses on findings from two pre-dividend African nations: Nigeria and Tanzania. These countries were selected because they are among the largest economies in Africa, in which the Gates Institute had local partnerships to facilitate the review of existing national policy documents.

Each sector of the Demographic Dividend Framework encompasses a wide array of strategic interventions, initiatives, or indicators that have proven effective for promoting per capita economic growth [16]. At the center of all sectors, the framework identifies *Governance and Economic Institutions* as a critical component to develop a favorable policy environment. Countries need to be politically stable, have solid institutions, and a favorable macroeconomic environment. While the *Governance and Economic Institutions* sector is an important component of the Demographic Dividend Framework, we focused our policy review on the remaining five sectors, given the cross-cutting nature of this sector.

The *Family Planning* input of the Demographic Dividend Framework addresses interventions that facilitate the realization of various benefits of family planning programs, accounting for the local cultural and social context while engaging local communities and promoting accessible and affordable modern contraceptive methods. The *Maternal and Child Health* input is focused on identifying interventions highlighting the importance of breastfeeding, safe motherhood initiatives, vaccinations, child nutrition programs, and case management of childhood illness. The *Education* input underscores interventions and policies that invest in universal primary education, ensure high quality education, and provide vocational training. The *Women’s Empowerment* sector highlights policies that prevent child marriage, protect women’s health, education, and human rights, and facilitate women’s access to the labor market. Finally, the *Labor Market* sector of the Demographic Dividend Framework includes initiatives to address unemployment rates, track and promote savings in various economic areas, encourage foreign investment, and develop the financial sector. More details for each of the sectors described, and their subsequent key strategic interventions, are described in detail elsewhere [16].

### Search strategy to review demographic dividend-favorable policies

The analytic framework to assess policy documents was based on the Demographic Dividend Framework. We identified relevant policy documents by following an inductive search strategy of open resources. We first searched for the most recent long-term vision plans and mid-term national strategic development plans issued by each country’s government that could be accessed online. In most cases, long-term plans covered ten years, while mid-term plans covered five years. We purposefully searched for long- and mid-term strategic planning documents and national policies developed by different sectors, reflecting the five inputs of the Demographic Dividend Framework on which we based our review (Fig. 1). We selected documents that were available and in use at the time of the policy review (2017/18). It is possible that some documents were in development and thus not available at the time of the review. Once we created a list of relevant policy documents, we consulted our local in-country collaborators via a self-designed survey, to validate the resulting list of policy documents and provide any additional documents that were missing from the list. Our in-country collaborators are experts from locally renowned institutions working in policy development, who are capable of providing their country-specific policy landscapes. All documents suggested by in-country collaborators were included if at least one sector of the Demographic Dividend Framework was covered in the documents.

Although global guidance specifies that pre-dividend countries should prioritize decreasing national fertility rates [15], we assessed policies developed for the Labor Market sector to determine each country’s readiness to support its burgeoning working age population. Finally, as previously mentioned, the Governance and Economic Institutions sector is considered an important input to maximize the demographic dividend. However, its cross-cutting nature impedes the ability to classify policies based on their individual impact. As such, we did not conduct a review of country policies relevant to Governance and Economic Institutions.

We reviewed the full text of each policy document from the final document list and extracted relevant text using a data extraction template that classified policies following different sectors of the Demographic Dividend Framework. We present an example of the template in Supplemental Figure A (see Additional file 1). The data extraction sheet was informed by the *Wheel of Prosperity*, which was developed by the Gates Institute in conjunction with the Demographic Dividend Framework, and provides a detailed breakdown of key interventions of each of the sectors of the Demographic Dividend Framework [16]. We organized the review of retrieved documents by policy phases including policy formulation, implementation or amendment [20]. The scope of this policy review was limited to the phase of policy formulation, which is regarded as bringing about acceptable actions to solve current issues identified on the policy agenda. Key information such as measurable targets, implementation timeline, success indicators, budget and reference pages were extracted from the identified official governments documents. The data extraction spread sheets were our primary outputs and were used for further analysis. We limited our extraction to documents written between 2010 and 2019. Several long- or mid-term documents were published in this time frame yet outlined implementation strategies that extended beyond this period (e.g., a 10-year long-term strategic plan may be implemented from 2016–2025).

We devised a three-category coding method using Harvey Balls to classify the extent to which the policy or strategy of a country is built towards each Demographic Dividend framework input [21]. The Harvey Ball icons correspond to the degree to which policies comprehensively addressed each intervention:



Policy documents were used to determine the policy-relevance of their content vis-à-vis the country demographic typology. A comprehensive policy category requires that the policy is specific (i.e., clear statements for regulations or plans to address a specific problem) and measurable (i.e., contains quantifiable measurement for expected results or targets). Being either not specific or not measurable, the policy is categorized as “Policy Mentions”. If there are no specific contents relating to the key intervention in the documents reviewed from the country’s full list, this intervention in the given country is determined as not mentioned in the reviewed documents.

Ethics approval for this study was waived by the institutional review boards of the Johns Hopkins Bloomberg School of Public Health in Baltimore, MD, USA and the Bill and Melinda Gates Foundation. This study was designated as non-human subjects research given its use of publicly available data.

## Results

We reviewed a total of 18 long-term and mid-term plan, sectoral strategy, and policy-specific documents from both pre-dividend nations, Nigeria and Tanzania. Country-specific documents identified as the fundamental materials for this policy review are listed in Table 1 and Supplemental document B1-4 (see Additional file 2). Overall, we identified policies that were conducive to attaining the demographic dividend in both countries. However, we observed a high degree of heterogeneity in how country-specific policies mention  or comprehensively address  each of the interventions of the Demographic Dividend Framework. Summary of country-specific findings by sector are provided in Table 2.

**Table 1:** Summary of documents reviewed in three pre-dividend African countries, as classified by the World Bank in 2015: Nigeria and Tanzania

	**Total number of documents**	**Long-term plan**	**Mid-term plan**	**Sector strategies**	**Specific/Other Policies**
*Nigeria*	8	Nigeria Vision 20:2020 Economic Transformation Blueprint	Economic Recovery & Growth Plan 2017–2020	4-Year Strategic Plan for the Development of the Education Sector 2011–2015 National Strategic Health Development Plan (NSHDP) 2010–2015 National Family Planning Communication Plan 2017	National Health Policy 2016 National Youth Policy 2019 National Employment Policy 2017
*Tanzania*	10	Tanzania Development Vision 2025	National Five-Year Development Plan 2016/17–2020/21	Health Sector Strategic Plan (HSSP IV) 2015–2020 Education Sector Development Programme 2008–17 National Family Planning Costed Implementation Plan 2019–2023 One Plan II: National Roadmap to Improve Reproductive, Maternal, Newborn, Child, and Adolescent Health 2016–2020	National Population Policy 2006 National Youth Development Policy 2007 National Employment Policy 2008 National Health Policy 2017

### Family planning

Nigeria and Tanzania provide policy mentions for two and three out of the four interventions of Family Planning, respectively. Family planning is mainly emphasized in Nigeria’s National Health Policy (NHP) 2016. The cultural and social contexts and cost reduction and product availability are integrated in the policy objectives and orientation section, but only limited to policy mentions. Benefits of family planning programs or community engagement are not covered. Specific policy orientation and initiatives address accounting for cultural and social context by promoting implementation of legislations that mitigate harmful cultural practices, such as female genital mutilation. Policy initiatives also emphasize reducing costs and ensuring product availability by promoting mechanisms to ensure access to quality reproductive health services, integration of reproductive maternal, neonatal, and child and adolescent health services.

**Table 2:**
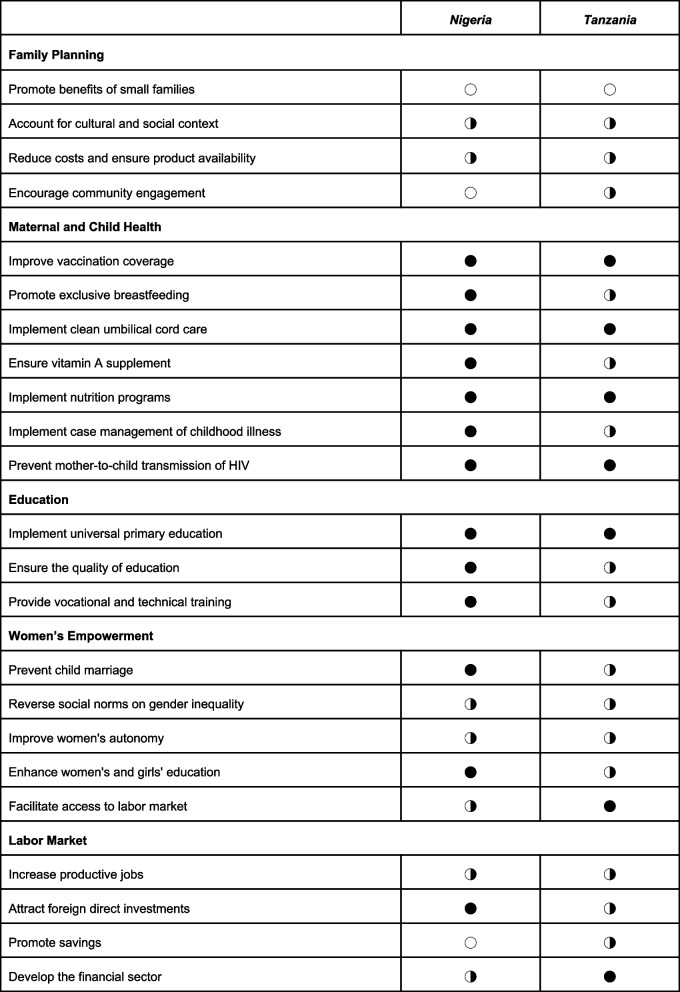
Summary of country-specific findings for Nigeria and Tanzania for five key policy sectors of the Demographic Dividend Framework and corresponding sector-specific components

In Tanzania, family planning is a core topic of the section of reproductive health in the 2006 revised National Population Policy, of which the goal is to coordinate and influence other policies, strategies, and programs that “*ensure sustainable development of the people and promote gender equality and the empowerment of women*”. Specifically, the Policy defined objectives with policy directions as follows:

- Account for cultural and social context by sponsoring public awareness of sexual and reproductive health and rights for all, promoting measures to eradicate harmful traditional practices (i.e., female genital mutilation), and encouraging men to participate in reproductive health programs;
- Reduce costs and ensure product availability by expanding quality, and strengthening delivery, of reproductive health services, including addressing problems such as infertility, cancers of the reproductive system, post-natal care, post abortion complications, and fistulae;
- Encourage community engagement by promoting and expanding quality reproductive health services and counselling for adolescents, men and women, and fostering participation and involvement of communities in provision of reproductive health services.

Reproductive health is further emphasized in Tanzania with special focus on strengthening the health system to provide quality services to contribute to ending preventable, maternal, newborn and child deaths and ensuring universal access to sexual, reproductive, and adolescent health services. Tanzania has clear targets for increasing uptake of family planning methods nationally from 27.4% to 60% in 2020 among married women (aged 15 to 49) and reducing adolescent fertility rates from 11.6% to less than 10% in 2020. The Plan also considers product availability and affordable prices, with emphasis on employing media campaigns and community outreach to improve adolescent and adult access to services.

### Maternal and child health

Nigeria comprehensively addresses all indicators of the Maternal and Child Health input of the Demographic Dividend Framework with two documents related to maternal and child health: the National Strategic Health Development Plan (NSHDP) and the National Health Policy (NHP). One of the strategic goals of the NSHDP 2010–2015 is to increase universal availability and access to an essential package of primary health care services with focus on particularly vulnerable groups including those of low socio-economic status and marginalized geographical areas. The essential package of care is grouped into three service delivery modes: “family-oriented, community-based services”; “population-oriented, schedulable services”; and “individually oriented clinical services”. The targeted elements of the framework include:

- Population oriented, scheduled routine immunizations to improve vaccination coverage;
- Family-oriented community-based services that promote breastfeeding practices;
- Policies that promote clean delivery and cord care;
- Population-oriented scheduled services to provide vitamin A supplementation for children under five;
- Family-oriented, community-based services to address malnutrition by supplementary feeding and follow-up management of severe acute malnutrition;
- A series of population-oriented, scheduled services that focus on preventing mother to child transmission of HIV via implementation of cotrimoxazole prophylaxis for HIV-positive mothers, adults, and children of HIV-positive mothers.

Nigeria further incorporates a system of accountability for routine immunization and ensuring vaccine security by establishing standards for injection safety and disposal and cold chain equipment; promoting comprehensive obstetrics care to reduce risks associated with pregnancy and childbirth; promoting awareness and strengthening the capacity of prevention, management and control of nutritional disorders with emphasis on facilitating community participation in nutrition interventions; promoting implementation of child survival strategies including provision of essential care services for the new-born; and providing universal access to comprehensive and quality HIV prevention, treatment care and support services.

Policies in Tanzania comprehensively cover four of the seven indicators: vaccination coverage, implementing clean umbilical cord care, implementing nutritional programs, and preventing mother-to-child transmission of HIV. In the Health Sector Strategic Plan 2015–2020 (HSSP IV), the Nutrition Services section and the Reproductive, Maternal, Newborn, Child and Adolescent Health section provide a set of strategies to improve maternal and child health in the five-year period of 2015–2020, guided by National Five-Year Development Plan and National Population Policy. Promotion of exclusive breastfeeding is briefly mentioned in reference to scaling up newborn, infant, and young child feeding services. At the community level, health programs aim to improve community mobilization, advocate utilization of the available services and stimulate families to seek early medical assistance. Specific initiatives and goals in each aspect are listed as follows:

- Improve vaccination coverage to over 90% in 90% of all districts by ensuring accessibility and utilization of immunization services through outreach and other programs.
- Implement clean umbilical cord care by increasing the proportion of skilled birth attendance to over 80% and continuing to expand provision of services during pregnancy, childbirth, and the post-natal period.
- Promote nutrition programs by ensuring food security at national and household levels and eradicating cultural barriers to improving community nutritional status. In addition, the *Essential Nutrition Action Approach* aims to reduce the percentage of underweight children from 16 to 11% in 2020, and to reduce the percentage of stunting children from 42 to 27% in 2020.
- Scale up application of a protocol to address integrated management of childhood illnesses, including building capacity of health care workers.
- Prevent mother-to-child transmission of HIV by ensuring all eligible patients receive Prevention of mother-to-child transmission (PMTCT) by 2020.

### Education

We assessed policies targeting the Education component of the Demographic Dividend Framework using three key indicators: implementing universal primary education, ensuring the quality of education, and providing vocational and technical training. Nigeria proposes national policies that effectively address all indicators of the Education input of the Demographic Dividend Framework. The importance of education is highlighted in Nigeria Vision 20:2020—the first pillar of Vision 20:2020 is guaranteeing the productivity and wellbeing of the people. The strategic orientation on education is to reform the educational system to enforce completion of mandatory nine-year universal basic education, while simultaneously building capacity in technical and vocational education.

Other national plans guarantee access to basic education for all and map out methods to improve the quality of secondary and tertiary education and encourages students to enroll in science, technology, engineering, and math (STEM) courses. Detailed strategies ensure quality of education by improving teacher quality, improving quality of education, and improving funding mechanisms to incentivize education performance and increase access. Strategies also provide vocational and technical training by building collaborative partnerships between partner, private, and state governments to establish vocational and technical institutes and increasing investment in STEM education.

Education policies are well-developed throughout Tanzania’s policy documents, while only primary education has specific targets under the Tanzania Development Vision 2025. The country has policy goals of implementing universal primary education by increasing net enrolment rates in primary school to 99% by 2012 and by providing special needs educational services to vulnerable children. Similarly, to Nigeria, additional Tanzanian policies entail ensuring quality education by providing satellite schools in remote areas and increasing the number of available teachers, educators, and instructors to reach optimal teacher to pupil ratios. Finally, Tanzania prioritizes providing vocational and technical training via strengthening knowledge and skills provision to children out of school, vulnerable groups (people with disabilities, orphans, people living with HIV/AIDS, elderly, young mothers, illiterate individuals, etc.), youths, and adult men and women in rural and urban areas.

### Women’s empowerment

Among the two countries, Nigeria and Tanzania provide policy mentions for all indicators of the Women’s Empowerment component of the Demographic Dividend Framework. Key indicators with additional comprehensive policy coverage include preventing child marriage and enhancing women’s and girls’ education (Nigeria) and facilitating women’s access to the labor market (Tanzania).

In Nigeria, prevention of child marriage is emphasized in the country’s national strategy, which specifies the age of marriage as 18 years old. Furthermore, the 18^th^ objective of the National Youth Policy of 2019 provides further protections for youth by advocating for and supporting the prevention of child marriage. Comprehensive policies to enhancing women’s and girls’ education included promoting equal access to basic education, designing, and organizing trainings for rural women to expand skill development, and establishing a National Empowerment Fund to endorse activities for women entrepreneurs. Key activities include constructing schools for girls in 13 states, constructing classroom blocks and schools that accommodate nomadic education-based learning across states.

Nigeria and Tanzania also mention policies encouraging women’s participation in local governance, ending or criminalizing harmful practices such as child marriage and/or female genital mutilation, and promoting equal access to basic education. Both countries incorporate elements of gender equity in national vision plans.

### Labor market

Nigeria and Tanzania provide policy mentions for all four indicators of the Labor Market. Key indicators with additional comprehensive policy coverage are “attracting foreign direct investments” (Nigeria) and “developing the financial sector” (Tanzania). Nigeria’s Economic Growth Recovery Plan 2017 emphasizes the importance of improving the capital account balance by attracting foreign capital into the economy, particularly foreign direct investment. This Plan aims to incentivize the inflow of foreign direct investment portfolio investments and remittances by approximately USD 7 billion by 2020. Nigeria’s National Employment Policy 2017 underscores job creation across various economic sectors, with special emphasis on creating and retaining jobs for vulnerable groups such as youth, women, and disabled persons, and ensuring greater participation of women in the workforce. This policy also strives to establish *Community Engagement Centers* across all governmental districts to aid job seekers in rural and urban communities. However, policies promoting job creation efforts or Community Engagement Centers are not described in measurable terms.

The policies of Tanzania comprehensively address developing the financial sector. The National Five-Year Development Plan 2016–2021 outlines “*new interventions to enable Tanzania industrialize in a way that will transform its economy and its society*”. The Plan describes a strategy to finance its agenda including (i) scaling up domestic revenue mobilization; (ii) increasing private sector participation; (iii) developing the domestic financial market; (iv) leveraging public sector resources to engage private sector in development projects; and (v) building strong debt management practices at the governmental level.

Tanzania also has policies promoting savings. Although not comprehensively addressed, Tanzania’s Vision 2025 emphasizes developing a national culture of saving and investing productively to generate wealth for individuals.

## Discussion

This policy analysis indicates that the study countries—Nigeria and Tanzania—have substantial policies in place to catalyze and accelerate the demographic transition and to accrue the potential benefits of the demographic dividend. Each country has adopted a multisectoral and integrated approach in developing strategies across health, education, economy, and gender equity sectors. Although these countries are different in terms of their economic and demographic characteristics, both have been classified as pre-dividend countries based on their TFR and projected share of working age population according to the demographic dividend typology developed by Ahmed and colleagues [22]. Estimates from 2014 indicated that the projected proportion of working age individuals would increase between 2015 and 2030 by 6.15 percentage points (p.p.) in Nigeria and by 6.70 p.p. in Tanzania, while the TFR of these countries was 5.41 and 4.92 children per woman in 2015, respectively. As a result, policies differ across countries, yet some themes arise.

Among the two countries, both have Family Planning policies that accounted for cultural and social contexts and that focused on ensuring access to quality family planning services, while maintaining affordable costs. These are key pillars for achieving high uptake of family planning services [23]. Countries could work to increase awareness of the implications of high population growth on national socio-economic development in efforts to promote the benefits of family planning programs and attain desired family size. This approach has been successfully integrated into national plans of other developing nations [14, 24]. Promoting the benefits of attaining a desired family size may lead to positive spillover effects in decreasing fertility rates and improving maternal and child health, education, and women’s empowerment, which can all work to accelerate the demographic transition in sub-Saharan Africa [25]. Government emphasis on family size, tailored to the available resources, should thus be considered when developing national policies.

Tanzania also mentions policies that encouraged community engagement in efforts to increase uptake of family planning services. Family planning programs have a stronger impact when the local cultural and social context is factored into policy development and community members are involved [10, 26]. This can be achieved by working with and training local community health workers, midwives, and community change agents. Encouraging community engagement can also facilitate community ownership and accountability, supporting the sustainability of strategies to improve family planning service delivery and provision. Moreover, engaging communities can foster positive shifts in community perception regarding family planning acceptability [27, 28]. Countries may consider supplementing existing policies with strategies that target community engagement to ensure reductions in fertility rates.

Nigeria and Tanzania comprehensively address, or mention, all indicators of the Maternal and Child Health input of the Demographic Dividend Framework. Both countries have comprehensive policies to improve child vaccination coverage. The differentiating factor is that Nigeria has specific and measurable policy directives and strategies in place to address all indicators of Maternal and Child Health. Although policy mentions are necessary, they are not sufficient to ensure that strategies are implemented as outlined in strategic and planning documents. Policies should be written with measurable goals at the design stage to ensure their success. Having specific and measurable policy goals also fosters a system of accountability and of making evidence-based decisions regarding resource allocation to address policy gaps.

Education policies generally focus on ensuring universal primary education by either increasing the availability and access of schools to all children, with particular focus on young girls and other vulnerable populations. Special attention is paid to hiring and training teachers, with an emphasis on teacher certification standards. Other policies include promoting education at secondary, university, and adult learning levels. Maximizing educational attainment and skill building at all levels, in particular for children and adolescents, is an integral component to reaping the full rewards of the demographic dividend. Some studies suggest that declines in fertility rates alone may not sufficiently maximize the demographic dividend without additional investments in education for the working age population [29]. Policies promoting educational attainment at all levels, in tandem with decreased fertility rates and job creation, have the potential to significantly boost human capital development and demographic dividends.

Relatedly, both countries have several policies targeting Women’s Empowerment. Many of these policies attempt to discourage harmful practices such as child marriage and female circumcision. Several also promote women’s participation in governance and have instated policies, protocols, and metrics to promote and track women’s engagement across different sectors. Supporting women’s and girls’ education are key factors in minimizing fertility rates and have been shown to affect other factors such as age at marriage, family size, and socio-economic status [27, 30]. However, as was the case for the Maternal and Child Health sector, neither country specified measurable policy initiatives.

Although not at the first tier of priority sectors for pre-dividend nations, as outlined in the Demographic Dividend Framework, we reviewed national documents for policies addressing the Labor Market. Meeting employment needs is critical to economic development, and in conjunction with family planning and education policies, can work to reduce the employment gap [10, 31]. Establishing policies that minimize unemployment levels today could help pre-dividend countries to ensure that the demographic dividend is successfully realized for future working individuals. In our review, policies stress job creation across various economic sectors, with emphasis on jobs for youth, women, and other vulnerable populations.

Both countries also outlined policies to attract foreign direct investments and develop the financial sector to support economic growth and the ability to enact policies as described. Based on our review, only Tanzania has policies that promote savings. The experience of East Asian tigers—namely South Korea, Taiwan, Hong Kong, and Singapore—suggests three mechanisms through which a changing age structure can promote economic growth, including increases in the labor force, the savings rate, and the investment rate [3]. Estimates across several high fertility rate countries, including 44 African nations, posit that increasing the working age share of the population by one percentage point, could simultaneously increase the savings share of the GDP, while reducing poverty [22]. Countries may consider incorporating more comprehensive policies that promote savings across various sectors.

### Limitations

Our policy review has some limitations. First, our review did not address policies relevant to local governance, a critical component of the Demographic Dividend Framework. Fostering good governance lays the foundations for establishing demographic dividend-favorable policies (Fig. 1) and is thus an integral element of capturing the demographic dividend. However, due to its cross-cutting nature, we opted to focus our review on policies that specifically target the other sectors of the Framework. Another limitation related to the Demographic Dividend Framework is that we narrowed our policy review into five sectors. It could be possible that other sectors are relevant for setting a favorable policy environment to harness a demographic dividend. However, the Gates Institute Demographic Dividend Framework was developed through a systematic literature review, which is why it contains similar sectors as other frameworks proposed to study a demographic dividend—such as Population Reference Bureau and the World Bank—but it expands prior work by considering the demographic dividend typology. For example, Population Reference Bureau proposed the four dividend framework, in which they identified education, health, economy, and governance and stability.

In addition, we may have concluded that policies addressing specific components of the Demographic Dividend Framework are not mentioned in national documents, while the strategies targeting these components are in place currently. Moreover, we did not focus our review on whether policies are being enforced. For instance, it is possible that activities related to specific components of Family Planning are taking place in-country but are not explicitly mentioned in national guidelines and planning documents. Similarly, we recognize that the years by which we bound our search criteria may have excluded policies that are being newly implemented or included policies that are out of date. While we acknowledge these limitations, we emphasize the important role that national documents play in ensuring that policies are implemented in accordance with a country’s goals and strategic plans, as well as their crucial role in guiding resource allocations. Existing documents provide a framework from which to identify and address policy gaps. Moreover, official documentation of policy strategies and targets creates a system of accountability and facilitates tracking of progress towards attaining and sustaining a demographic dividend-favorable policy environment.

Finally, an integral element of ensuring that the demographic dividend is fully realized, is investing in resources to improve data collection and to facilitate access and availability to quality data for decision making purposes. Our review process does not evaluate implementation of country commitments. Doing so requires establishment of monitoring and evaluation systems to track inputs and progress towards policy goals, an important component of the policy-implementation cycle [16]. Although some countries specified monitoring and evaluation plans in conjunction with national policy frameworks, this was not uniformly the case. Policy makers and development partners may wish to develop monitoring and evaluation plans to support policy implementation efforts and assess progress towards intended goals. Additionally, countries could expand research and data analysis endeavors to inform policy and program decision-making and guide monitoring and evaluation efforts. Considerations may also be made for disseminating results and allowing for policies and implementation plans to adjust because of monitoring and evaluation and/or research findings.

### Strengths

A unique strength of this policy review is its focus on two pre-dividend African nations. Previous policy reviews have been all-encompassing in nature and not specific to countries undergoing different stages in their demographic transition [13, 14, 32]. By restricting our policy review to specific nations, we can highlight demographic dividend favorable policies that are specific to each pre-dividend country. Second, we systematically reviewed countrywide planning documents developed by national stakeholders using a holistic framework that encompasses various potential benefits from a demographic dividend beyond economic benefits. This framework presents several other benefits compared to other existing demographic dividend frameworks as described by Cardona and colleagues [16]. Finally, we believe that our strong working partnerships with local collaborators facilitated the identification and review of key documents in each of these two countries. The collaborative nature of our review can also foster local ownership of study findings. Through this review process, key stakeholders may be able to better advocate for policies or allocate resources to address policy gaps.

Taken together, we recommend that Nigeria and Tanzania, two pre-dividend nations, (1) expand targeted policies and initiatives to address family planning, with emphasis on promoting the benefits of these programs and of attaining the desired family size, and fostering community engagement, (2) incorporate policies that further women’s empowerment across all sectors, and (3) ensure that all policies are specific and measurable, with special focus on policies aimed at improving maternal and child health, maximizing educational attainment, and advancing women’s empowerment. In addition, both countries may consider policies that improve the quality of education and training of youth to respond to future labor market needs. It is our hope that other pre-dividend nations and development partners may use our methods to conduct similar policy reviews and possibly to implement a series of policy amendments to capitalize on their demographic dividend potential.

## Conclusion

As African countries recommit to improving socio-economic development and move forward with implementing demographic dividend roadmaps and strategies, it is important to continuously review existing policies for their relevance. We developed a systematic and simple way to comprehensively assess the policy environment of two pre-dividend nations, with specific consideration towards identifying demographic dividend-favorable policies. Through our policy review, we identified strategic policy amendments that both pre-dividend countries could undertake to capitalize on their demographic dividend. Countries with similar demographic structures may consider conducting similar review to close existing policy gaps and accrue the full potential of the demographic dividend. By ensuring the establishment of prudent and timely policies that promote health, education, gender equity, and well-functioning financial and labor markets, African countries can realize a demographic dividend and uphold sustainable levels of economic growth and development.

## Supplementary Information

**Additional file 1.** Data extraction template for the review of national policy documents.

**Additional file 2.** List of policy documents reviewed by country: Nigeria (B1) and Tanzania (B2).

**Additional file 3. **Review of national policy documents from Nigeria.

**Additional file 4. **Review of national policy documents from Tanzania.

## Data Availability

The data supporting the conclusions of this article are publicly available as government policy documents. We list all policy documents reviewed for this manuscript in Additional file 2. Data abstracted from each policy document are available in Additional files 3 and 4.

## Abbreviations

PRF: Population Reference Bureau
TFR: Total fertility rate
NHP: Nigeria National Health Policy
NSHDP: Nigeria National Strategic Health Development Plan
HSSP IV: Tanzania Health Sector Strategic Plan 2015–2020
PMTCT: Prevention of mother-to-child transmission
STEM: Science, technology, engineering, and math

## References

[1] AU Roadmap on harnessing the demographic dividend through investments in youth. Assembly of the Union Twenty-Sixth Ordinary Session 30–31 January 2016, Addis Ababa, Ethiopia; 2017. Retrieved from https://library.au.int/au-roadmap-harnessing-demographic-dividend-through-investments-youth.

[2] Bloom DE, Finlay JE (2009). Demographic change and economic growth in Asia.

[3] Bloom DE, Williamson JG (1998). Demographic transitions and economic miracles in emerging Asia.

[4] Cooper RN, Bloom DE, Canning D, Sevilla J (2003). The demographic dividend: a new perspective on the economic consequences of population change.

[5] Eastwood R, Lipton M (2011). Demographic transition in sub-Saharan africa: how big will the economic dividend be?.

[6] Bloom DE, Canning D, Fink G, Finlay JE (2007). Does age structure forecast economic growth?.

[7] Reher DS (2011). Economic and social implications of the demographic transition.

[8] Lesthaeghe R (2010). The unfolding story of the second demographic transition.

[9] Van De Kaa DJ (1987). Europe’s second demographic transition.

[10] Bloom DE, Humair S, Rosenberg L, Sevilla JP, Trussell J (2013). A demographic dividend for sub-Saharan Africa: source, magnitude, and realization.

[11] Bloom DE, Canning D, Fink G, Finlay J (2010). Realising the demographic dividend: is africa any different?.

[12] Bloom DE, Kuhn M, Prettner K (2017). Africa’s prospects for enjoying a demographic dividend.

[13] Patierno K, Gaith S, Leahy Madsen E (2009). Which policies promote a demographic dividend? An evidence review.

[14] Shekar M, Yazbeck A, Hasan R, Bakilana A (2016). Population and development in the Sahel: policy choices to catalyze a demographic dividend.

[15] Achieving the demographic dividend: an operational tool for country-specific investment decision-making in pre-dividend countries. The World Bank. 2019. Retrieved from https://documents1.worldbank.org/curated/en/781891550815372274/pdf/Demographic-Dividend-Operational-Tool-for-Pre-Dividend-Countries.pdf.

[16] Cardona C, Rusatira JC, Cheng X, Silberg C, Salas I, Li Q, Bishai D, Rimon JG. Generating and capitalizing on the demographic dividend potential in sub-Saharan Africa: a conceptual framework from a systematic literature review. Gates Open Res. 2020;4:145. 10.12688/gatesopenres.13176.1 PMC8028847 33870102

[17] Weny K, Snow R, Zhang S. The demographic dividend atlas for Africa: Tracking the potential for a demographic dividend. UNFPA. 2017. Retrieved from www.unfpa.org.

[18] Canning D, Raja S, Yazbeck AS. Africa's demographic transition: dividend or disaster? The World Bank Publications; 2015.

[19] Global monitoring report 2015/2016: Development goals in an era of demographic change: The World Bank. 2015. Retrieved from https://openknowledge.worldbank.org/entities/publication/72b4e4d6-007c-53b1-a28f-2b32cd64a80e.

[20] Bradham DD (1985). Health policy formulation and analysis.

[21] Ouedraogo O, Doudou MH, Drabo KM, Garnier D, Zagré NM, Sanou D, Reinhardt K, Donnen P (2020). Policy overview of the multisectoral nutrition planning process: the progress, challenges, and lessons learned from Burkina Faso.

[22] Ahmed SA, Cruz M, Quillin B, Schellekens P (2016). Demographic change and development: looking at challenges and opportunities through a new typology.

[23] Haider T, Sharma M (2012). Barriers to family planning and contraception uptake in sub-saharan africa: a systematic review.

[24] McKelvey C, Thomas D, Frankenberg E (2012). Fertility regulation in an economic crisis.

[25] Family planning promotes the demographic dividend. Global Leaders Council for Reproductive Health, Aspen Global Health and Development at the Aspen Institute. 2011. Retrieved from https://www.aspeninstitute.org/wp-content/uploads/files/content/docs/pubs/Demographic%20Dividend%20Policy%20Brief[1]_nov27.pdf.

[26] Recent fertility trends in sub-Saharan Africa: Workshop Summary. Committee on Population; Division of Behavioral and Social Sciences and Education. Washington, DC: National Academies of Sciences, Engineering, and Medicine; 2016. 27010050

[27] Majumder N, Ram F (2015). Explaining the role of proximate determinants on fertility decline among poor and non-poor in Asian countries.

[28] Bongaarts J, Watkins SC (1996). Social interactions and contemporary fertility transitions.

[29] CrespoCuaresma J, Lutz W, Sanderson W (2014). Is the demographic dividend an education dividend?.

[30] Shapiro D (2012). Women’s education and fertility transition in sub-saharan Africa.

[31] Guengant JP, May JF (2013). African demography.

[32] Africa and the challenge of realizing the demographic dividend. Population Reference Bureau; Africa Union Commission. 2013. Retrieved from https://www.prb.org/wp-content/uploads/2013/04/africa-demographicdividend-challenges.pdf.

