# Supplementary figures and images for: Demographic dividend-favorable policy environment in two pre-dividend African nations: review of national policies and prospects for policy amendments in Nigeria and Tanzania
Source: BMC Public Health. 2023 Jun 5;23:1070. doi: 10.1186/s12889-023-15690-z (PMC10240741; doi:10.1186/s12889-023-15690-z)

1. **Data extraction template for the review of national policy documents**

**
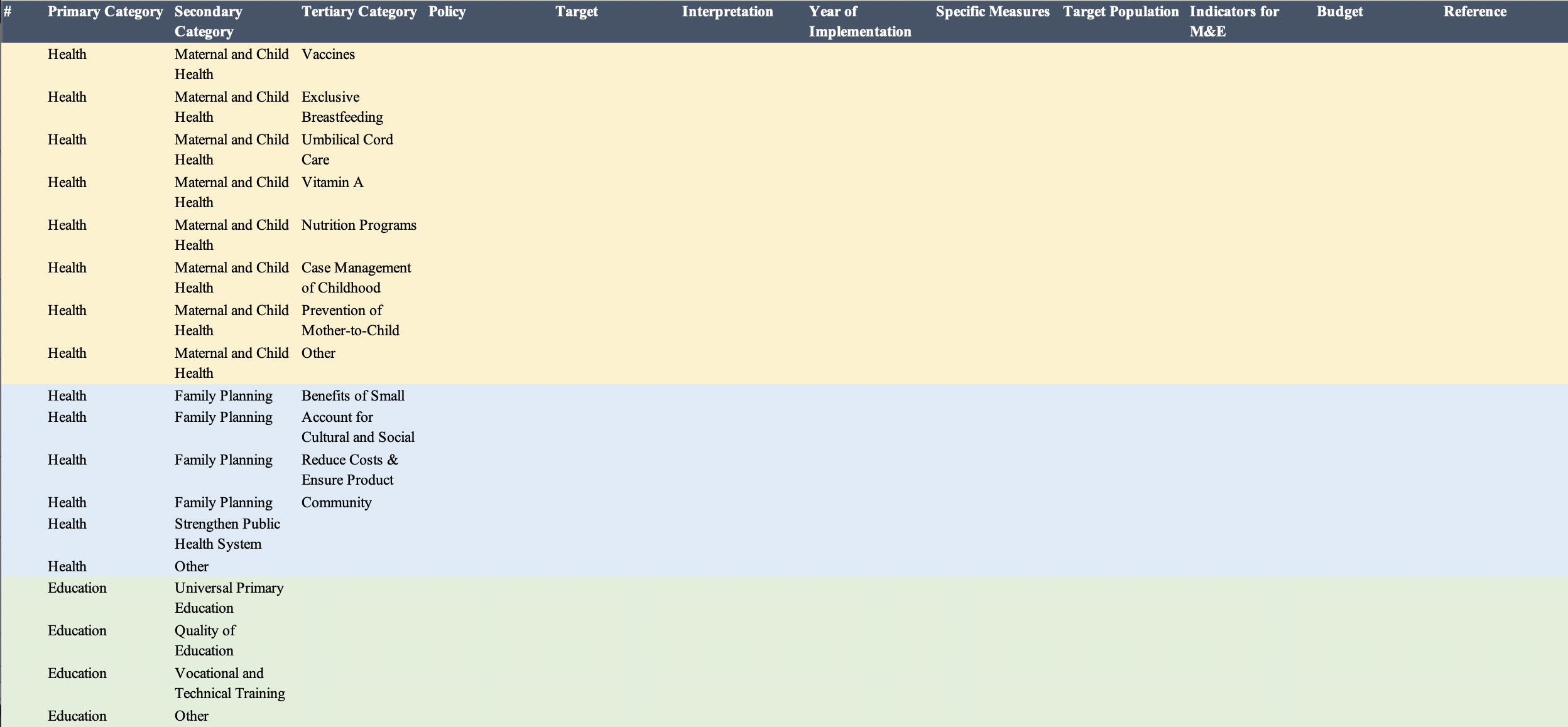
**

Supplement: Supplementary file 1 — Additional file 1. Data extraction template for the review of national policy documents. [file 12889_2023_15690_MOESM1_ESM.docx]
