# Supplementary material for: Demographic dividend-favorable policy environment in two pre-dividend African nations: review of national policies and prospects for policy amendments in Nigeria and Tanzania
Source: BMC Public Health. 2023 Jun 5;23:1070. doi: 10.1186/s12889-023-15690-z (PMC10240741; doi:10.1186/s12889-023-15690-z)
Supplement: Supplementary file 2 — Additional file 2. List of policy documents reviewed by country: Nigeria (B1) and Tanzania (B2). [file 12889_2023_15690_MOESM2_ESM.docx]

**B. List of policy documents reviewed by country: Nigeria (B1) and Tanzania (B2)**

**B1. Nigeria**

*Nigeria Vision 20:2020 Economic Transformation Blueprint.* Federal Republic of Nigeria: National Planning Commission. Nigeria: December 2009.

*Economic Recovery & Growth Plan 2017-2020*. Federal Republic of Nigeria: Ministry of Budget & National Planning. Nigeria: 2017.

*4-Year Strategic Plan for the Development of the Education Sector 2011-2015.*Federal Republic of Nigeria: Federal Ministry of Education. Abuja, Nigeria: 2012.

*National Strategic Health Development Plan (NSHDP) 2010-2015*. Federal Republic of Nigeria: Federal Ministry of Health. Abuja, Nigeria: 2010.

*National Family Planning Communication Plan 2017*: Strategy for Increasing the use of Modern Contraceptives in Nigeria. Federal Republic of Nigeria: Ministry of Health. Abuja, Nigeria: 2017.

*National Health Policy 2016: Promoting the Health of Nigerians to Accelerate Socio-economic Development*. Federal Republic of Nigeria: Federal Ministry of Health. Abuja, Nigeria: 2016.

*National Youth Policy 2019: Enhancing Youth Development and Participation in the context of Sustainable Development*. Federal Republic of Nigeria: Federal Ministry of Youth and Sports Development. Abuja, Nigeria: 2019.

*National Employment Policy 2017*: Objectives, Measures, Strategies and Institutional Framework to Meet the Challenges of Rising Unemployment and Underemployment. Federal Republic of Nigeria: Ministry of Labour and Employment. Abuja, Nigeria: 2017.

**B2. Tanzania**

*The Tanzania Development Vision 2025.* The United Republic of Tanzania: Planning Commission. Tanzania: 2000.

*National Five-Year Development Plan 2016/17-2020/21.* The United Republic of Tanzania: Ministry of Finance and Planning. Tanzania: 2016.

*Education Sector Development Programme 2008-17.* The United Republic of Tanzania: Ministry of Education. Tanzania: 2008.

*Health Sector Strategic Plan (HSSP IV) 2015-2020: Reaching all Households with Quality Health Care.* The United Republic of Tanzania: Ministry of Health and Social Welfare. Tanzania: 2015.

*National Population Policy.* The United Republic of Tanzania: Ministry of Planning, Economy and Empowerment. Tanzania: 2006.

*National Family Planning Costed Implementation Plan 2019-*2023: The United Republic of Tanzania: Ministry of Health, Community Development, Gender, Elderly and Children. Dar es Salaam, Tanzania: 2019.

*One Plan II:* National Roadmap to Improve Reproductive, Maternal, Newborn Child, and Adolescent Health 2016-2020. The United Republic of Tanzania: Ministry of Health, Community Development, Gender, Elderly and Children. Dar es Salaam, Tanzania: 2016.

*National Youth Development Policy.* The United Republic of Tanzania: Ministry of Labour, Employment and Youth Development. Tanzania: December 2007.

*National Employment Policy.* The United Republic of Tanzania: Ministry of Labour, Employment and Youth Development. Dar es Salaam, Tanzania: 2008.

*National Health Policy 2017.* The United Republic of Tanzania: Ministry of Health, Community Development, Gender, Elderly and Children. Dar es Salaam, Tanzania: 2016.
